# Purine but Not Pyrimidine De Novo Nucleotide Biosynthesis Inhibitors Strongly Enhance the Antiviral Effect of Corresponding Nucleobases Against Dengue Virus

**DOI:** 10.3390/molecules30020210

**Published:** 2025-01-07

**Authors:** Laurent F. Bonnac, Christine D. Dreis, Madhu Rai, Robert J. Geraghty

**Affiliations:** Center for Drug Design, College of Pharmacy, University of Minnesota, Minneapolis, MN 55455, USA

**Keywords:** broad-spectrum antivirals, nucleobases, drug combination, flaviviruses

## Abstract

Every year, dengue virus affects hundreds of millions of individuals worldwide. To date, there is no specific medication to treat dengue virus infections. Nucleobases, the base of a nucleoside without ribose, are understudied as potential treatments for viral infections. Antiviral nucleobases are converted in infected cells to their corresponding nucleoside triphosphate active form. Importantly, the conversion of nucleobases to their active nucleotide form and their antiviral effect can be enhanced when combined with de novo nucleotide biosynthesis inhibitors. In this work, we evaluated seven purine and pyrimidine nucleobases alone or combined with six purine or pyrimidine de novo nucleotide biosynthesis inhibitors, including novel prodrugs. Our study revealed that while a strong potentiation of purine nucleobases by purine de novo nucleotide biosynthesis inhibitors was observed, the pyrimidine nucleobases were not potentiated by pyrimidine de novo nucleotide biosynthesis inhibitors, possibly highlighting a significant difference between the modulation of purine versus pyrimidine de novo pathways and their impact on nucleobase potentiation. Most significant antiviral effects and potentiation were observed for Favipiravir, T-1105, and ribavirin nucleobases combined with purine nucleotide de novo synthesis inhibitors. These results are significant because drug combinations may solve the limited efficacy observed for some antiviral nucleobase drugs such as Favipiravir.

## 1. Introduction

Dengue virus (DENV) is a global health threat that affects hundreds of millions of individuals each year, including approximately 500,000 severe cases and 22,000 deaths (WHO). The limited effectiveness of vaccines to prevent DENV infection is partly due to the necessity to generate potent immune responses against the four dengue serotypes. There is an urgent need for therapies to combat DENV with record high number of cases in 2024 and the spread of DENV to new geographic areas in Europe and America. 

**Figure 1:**
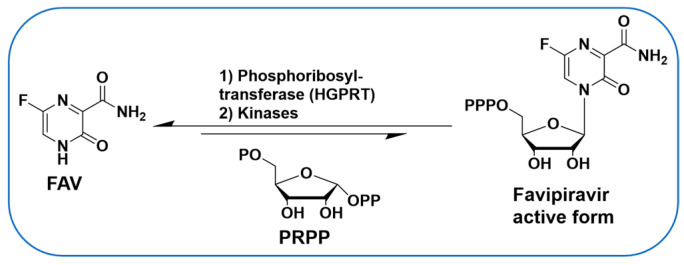
FAV is converted in cells to its nucleoside triphosphate form.

**Figure 2:**
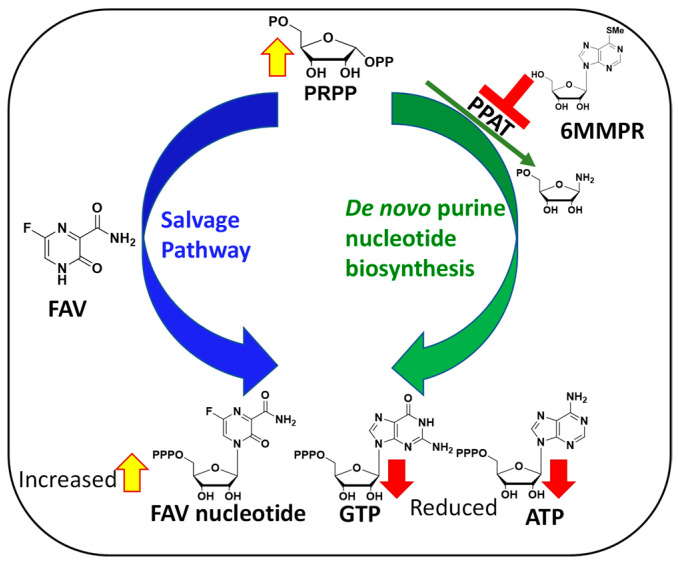
Mechanism behind FAV/6MMPR antiviral synergy.

Antiviral nucleoside analogues have been studied extensively and represent some of the most successful molecules to target viruses. However, limited studies have been carried out on nucleobases (the base of a nucleoside without ribose) to target viruses. A notable exception is the well-studied Favipiravir (FAV, Figure 1), a purine nucleobase analogue and a broad-spectrum antiviral drug [1,2,3,4]. In DENV-infected cells, purine nucleobases such as FAV can be converted in one step to their corresponding nucleoside monophosphate form by hypoxanthine-guanine phosphoribosyltransferase (HGPRT) (Figure 1) [5], thereby offering an alternative to antiviral nucleosides in which conversion to the monophosphate is often rate-limiting [6,7,8]. Nucleobases have been used to target DENV and other viruses [9] and used via a synergistic and broad-spectrum potentiation strategy combining the purine nucleobase analogue FAV with the purine de novo nucleotide biosynthesis inhibitor 6-methylmercaptopurine riboside (6MMPR) (Figure 2) [10].

The mechanisms behind FAV potentiation by 6MMPR that leads to a synergistic and broad-spectrum antiviral activity against severe acute respiratory syndrome coronavirus 2 (SARS-CoV-2), influenza A virus, Zika virus (ZIKV), and human coronavirus OC43 has been explained as follows. Briefly, 6MMPR inhibits phosphoribosyl-pyrophosphate amidotransferase (PPAT) responsible for the first committed step of the de novo purine nucleotide biosynthesis [10]. PPAT inhibition leads to two important outcomes. First, PPAT inhibition lowers the concentration of endogenous ATP and GTP in infected cells able to compete with the active form of FAV for use by the viral RNA-dependent RNA polymerase (RdRp). Second, PPAT inhibition increases the phosphoribosyl-pyrophosphate (PRPP) concentration, thereby increasing FAV conversion to its active form via the purine salvage pathway (Figure 2).

On this basis, we performed additional studies to increase the scope and efficacy of our potentiation strategy. Critically, both the de novo purine and pyrimidine nucleotide biosynthesis pathways are upregulated in virus-infected cells and thus offer broad-spectrum targets to selectively potentiate antiviral nucleobases [11]. In addition, both purine and pyrimidine nucleobases can be converted in one step to their corresponding mononucleotide through the nucleotide salvage pathway, including via adenine, hypoxanthine, guanine phosphoribosyltransferases for purines, and uracil and orotate phosphoribosyltransferases for pyrimidines. Up to now, we have only demonstrated the efficacy of our potentiation approach with the purine nucleobase analogues FAV and analogue T-1105 potentiated by 6MMPR. Other purines need to be tested, and improvements upon 6MMPR toxicity are desirable. Lastly, the nucleobase potentiation strategy has yet to be studied for antiviral pyrimidine analogues co-administered with pyrimidine nucleotide de novo biosynthesis inhibitors. Therefore, the main objectives of the research presented here are as follows: (1) to investigate 6MMPR potentiation of further purine nucleobases, (2) to determine if novel 6MMPR prodrugs can potentiate FAV antiviral activity, and (3) to establish if our potentiation strategy applies to pyrimidine nucleobases when combined with pyrimidine de novo pathway inhibitors. Herein, we present the evaluation of a variety of purine and pyrimidine nucleobase analogues alone or combined with corresponding de novo purine or pyrimidine nucleotide biosynthesis inhibitors in a DENV replicon assay.

## 2. Results

We initiated these studies with the evaluation of a library of pyrimidine and purine nucleobases, alone or in combination with respective pyrimidine or purine de novo nucleotide biosynthesis inhibitors as potentiators. We have already shown that purine de novo nucleotide biosynthesis inhibitors can potentiate the activity of purine nucleobases [10], and we were interested to see if pyrimidine de novo nucleotide biosynthesis inhibitors could potentiate pyrimidine nucleobases.

**Figure 3:**
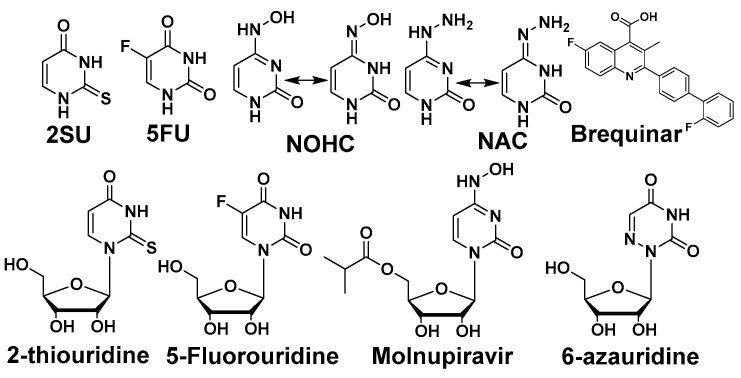
Pyrimidine nucleobase and nucleoside analogues and potentiators.

**Figure 4:**
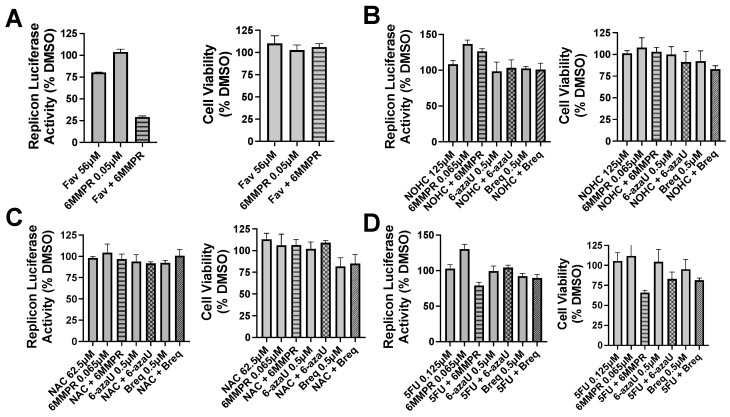
Nucleobase and anti-metabolite combinations and effects on DENV replicon replication and cell viability. Bars for single compound treatment are gray. Stippled or hatched bars represent combination treatments. DENV BHK replicon cells were treated with compound plus vehicle (DMSO) or two compounds and evaluated for luciferase activity (left graph) and cell viability (right graph). (**A**) Single non-toxic and minimally active doses of Fav and 6MMPR or combination. (**B**) NOHC alone or combined with 6MMPR, 6-azaU, and Breq. (**C**) NAC alone or combined with 6MMPR, 6-azaU, and Breq. (**D**) 5FU alone or combined with 6MMPR, 6-azaU, and Breq. Breq = Brequinar.

**Figure 5:**
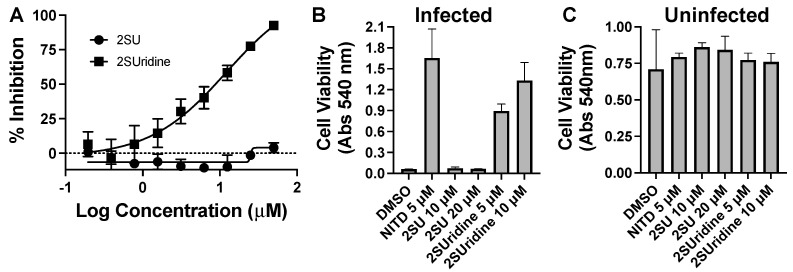
Flavivirus inhibition by the nucleobase 2-thiouracil (2SU) and the corresponding nucleoside 2-thiouridine (2SUridine). (**A**) Dose–response assay for inhibition of a ZIKV nanoluc reporter. Cells were inoculated with reporter virus for two hours, washed, and then compound-added. Three days later, cells were lysed, and the luciferase activity was analyzed. (**B**) DENV-induced cytopathic effects in compound-treated cells. Cells were inoculated with DENV for two hours, washed, and compound-added for five days. A neutral red assay was performed to determine cell viability. (**C**) Same as in (**B**), except the cells are not treated with virus. NITD = NITD008 a positive-control DENV inhibitor.

Pyrimidine and potentiators studies: We chose four known pyrimidine nucleobase analogues, 2-thiouracil (2SU), 5-fluorouracil (5FU), *N*-hydroxy-cytidine (NOHC), and *N*-amino-cytidine (NAC) (Figure 3). The corresponding nucleosides (2-thiouridine, 5-fluorouridine, and Molnupiravir; Figure 3) of nucleobases (2SU, 5FU, and NOHC) are known to display antiviral activities [12,13,14]. For the pyrimidine de novo nucleotide biosynthesis inhibitors (potentiators), we selected 6-azauridine, which inhibits uridine monophosphate synthase and Brequinar, a dihydroorotate dehydrogenase inhibitor (Figure 3). None of the four pyrimidine nucleobases displayed clear activity on their own when tested using a DENV replicon cell line (Table 1). We were interested to see if a potentiation strategy (i.e., blocking de novo pyrimidine nucleotide biosynthesis) could generate antiviral activity for the four pyrimidine nucleobases. The co-treatment with the anti-metabolites 6-azauridine or Brequinar did not induce any anti-replicon inhibition for a high non-toxic dose of any of the four pyrimidine nucleobases (Figure 4B–D). This result contrasts with the clear enhanced effect observed when a purine nucleobase (FAV) was combined with purine nucleotide synthesis inhibitor 6MMPR (Figure 4A). Control inhibitors 2-thiouridine and Molnupiravir were used for their described antiviral activities as pyrimidine nucleoside analogues. While 2-thiouridine has been published to inhibit DENV and Zika virus (ZIKV) [12], we did not observe activity for 2-thiouridine in the DENV BHK replicon cell line (Table 1). We did, however, observe antiviral activity for 2-thiouridine, but not 2-SU, in assays measuring DENV-induced cytopathic effects or ZIKV reporter virus replication, indicating antiviral activity but not anti-replicon activity (Figure 5).

**Figure 6:**
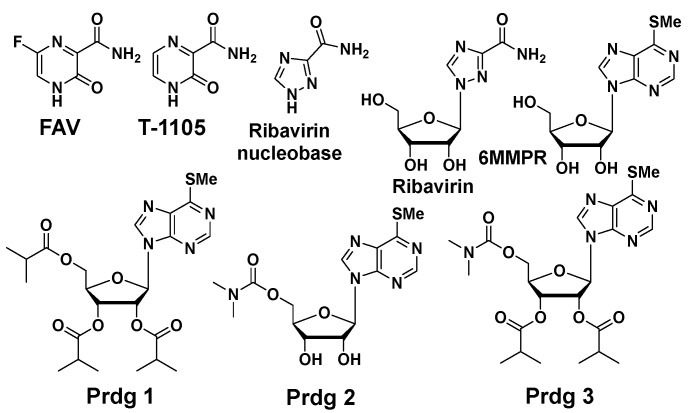
Purine nucleobases, nucleosides, and potentiators/prodrugs.

**Figure 7:**
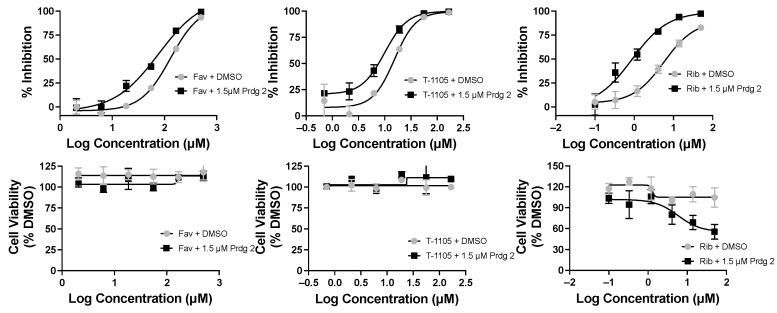
Favipiravir (Fav), T-1105, and ribavirin base (Rib) combined with 6MMPR prodrug (Prdg 2) effects on DENV replicon replication. DENV BHK replicon cells were treated with compounds indicated and three days later evaluated for luciferase activity and cell viability. The results for nucleobase plus vehicle (DMSO) were plotted with the corresponding Fav, T-1105, or Rib with 1.5 μM Prdg 2. Fav doses ranged from 500 to 2 μM; T-1105 doses ranged from 167 to 0.7 μM. Rib doses ranged from 50 to 0.1 μM. Results were plotted in GraphPad Prism, and the EC_50_/CC_50_ values are listed in Table 3.

Purines and potentiators studies: For the purines, we chose FAV and its defluorinated analogue T-1105, as well as the ribavirin nucleobase (Figure 6), which we first described to have a strong yet non-cytotoxic anti-DENV activity [9]. For the purine de novo nucleotide biosynthesis inhibitors (potentiators), we selected 6MMPR (Figure 6), based on earlier studies [10], as well as three novel 6MMPR prodrugs synthesized in 1–2 steps via simple chemical procedures (see Supplementary Materials). We developed the novel 6MMPR prodrugs (Figure 6) to address 6MMPR’s cytotoxicity and each showed improved cell culture viability compared to parent 6MMPR (Table 2). We first tested 6MMPR and prodrugs for their ability to potentiate the anti-replicon activity of FAV. Dose–response analysis of FAV with a single non-toxic dose of potentiator resulted in a shift in the EC_50_ curve and a reduction in the EC_50_ value compared to FAV dose–response with vehicle DMSO (Figure 7 and Figure S1, Table 3). T-1105 was also strongly potentiated by 6MMPR and prodrugs (Figure 7 and Figure S2, Table 3), demonstrating a broader applicability of our strategy to two different purine nucleobase analogues. The combination of ribavirin nucleobase and potentiators conferred some toxicity not shown by either compound alone (Figure 7 and Figure S3, Table 3) and in contrast to what was observed for the other purine nucleobases.

## 3. Discussion

Broad-spectrum antivirals are urgently needed to develop countermeasures against emerging and re-emerging viruses [15,16]. Developing broad-spectrum antivirals allows for accelerated drug approval compared to the classical virus-specific drug development which is too slow and costly to provide solutions in possible pandemic emergencies. Only a few broad-spectrum antiviral drugs are currently approved, and these have some limitations. FAV is a broad-spectrum antiviral drug active against many viruses, but it has displayed limited efficacy in clinical trials against Ebola, SARS-CoV-2, and influenza viruses [17,18,19,20,21]. One limitation of FAV is that it can be a poor substrate for HGPRT, the enzyme responsible for its conversion to its corresponding nucleoside monophosphate [5]. Our drug combination strategy is designed to significantly increase the formation of FAV’s active form, leading to a synergistic and broad-spectrum antiviral effect via two distinct yet complementary mechanisms [10].

In this study, we expand the scope of nucleobases and potentiators targeting DENV as a flavivirus model. The lessons learned from the study presented here are multiple. First, we did not observe the potentiation of pyrimidine nucleobases with a de novo pyrimidine nucleotide biosynthesis inhibitor. The reasons behind the lack of potentiation for pyrimidines are unclear; however, some hypotheses can be formed. The inhibition of the pyrimidine nucleotide de novo pathway may not result in a significant buildup of the PRPP concentration needed to increase the formation of the active antiviral forms. While the conversion of 5FU and 2SU is known to proceed naturally to the corresponding nucleotide in cells, the conversion of NOHC and NAC has not been described. Similarly to Molnupiravir, NOHC and NAC exist as tautomeric forms, allowing them to mimic U or C analogues. Yet, if NOHC and NAC mostly exist as C analogues in cells, the conversion to their corresponding nucleotide may not be possible. Indeed, phosphoribosyltransferases of the pyrimidine salvage pathway only convert uracil or orotate analogues but not cytidine analogues. Lastly, the concentration of endogenous pyrimidine nucleotide in cells may not be affected as much to observe a potentiation effect.

We were surprised at the lack of activity for nucleoside 2-thiouridine in the DENV replicon cells. 2-Thiouridine is an inhibitor for a variety of viruses, including DENV, probably by targeting RNA-dependent RNA polymerase [12], so we expected robust anti-replicon activity. The replicon cells were constructed using the BHK cell line, and others have shown antiviral activity for 2-thiouridine in BHK cells, so it is unlikely that there is a defect in converting 2-thiouridine to its active nucleoside triphosphate form unless that defect arose during the construction of the replicon cell line. Alternatively, the target for 2-thiouridine may be missing or unimportant in the replicon cell line, such as some aspect of virus assembly or possibly viral methyltransferase activity.

For the purine studies, the discovery that ribavirin nucleobase can be potentiated through our strategy is particularly interesting. Ribavirin nucleobase is considerably less cytotoxic than ribavirin, yet it possesses a similarly strong antiviral activity [9]. This result is significant because ribavirin nucleobase may represent a realistic alternative to FAV and a possible alternative to ribavirin nucleoside itself, which induces undesirable side effects in patients.

## 4. Materials and Methods

### 4.1. Cells and Viruses

The following cell lines were used in this study: (i) Huh7 was purchased from JCRB Cell Bank (Seikisui XenoTech, Kansas City, MO, USA) and cultured in Minimum Essential Medium (MEM) supplemented with 10% fetal bovine serum (FBS), and 1× non-essential amino acids (NEAA), 1 mM sodium pyruvate, 100 IU penicillin/streptomycin per mL, 5 µg/mL plasmocin (InvivoGen, San Diego, CA, USA), 1× GlutaMAX, and (ii) BHK pD2-hRucPac-2ATG30 DENV replicon cells were a gift from M. Diamond (Washington University School of Medicine) and cultured in Dulbecco’s modified Eagle’s medium (DMEM) supplemented with 10% FBS, 100 IU penicillin/streptomycin per mL, 5 µg/mL plasmocin, 1× GlutaMAX, and 3 µg/mL puromycin (InvivoGen). (iii) A549 Ace2 TMPRSS2 cells [22] were provided by M. Saeed (Boston University) and grown in DMEM supplemented with 10% FBS, 100 IU penicillin/streptomycin per mL, 10 mM HEPES, 1× NEAA, 1× GlutaMAX, 1 mM sodium pyruvate, 5 µg/mL plasmocin, 0.5 µg/mL puromycin, and 0.5 µg/mL blasticidin (InvivoGen).

The following viruses were used in this study: (i) ZIKV PAN1 Nluc reporter virus has a nanoluc gene inserted into the viral genome as described [23]. The virus was propagated and virus preps tittered as described [23]. (ii) DENV type 2 New Guinea C strain (VR-1584) was obtained from ATCC (Manassas, VA, USA) and propagated in C6/36 mosquito cells as described [9].

### 4.2. Cell Viability Assay (MTS)

Cell viability assays were performed as described in [9,10,23]. Briefly, DENV replicon cells were plated at 1500–2000 cells per well of a 96-well dish plate (Corning, NY, USA) the day before use. The next day, compounds were added and cells incubated at 37 °C, 5% CO_2_, in a humidified incubator for three days. The cells were washed with phosphate-buffered saline (PBS) and incubated with the MTS reagent (Promega G5430, Madison, WI, USA) as per the manufacturer’s instructions. The plates were read at a 490 nm wavelength at 30 min intervals in a BioTek (Winooski, VT, USA) Neo2 plate reader.

### 4.3. DENV Replicon Cell Assay

The DENV replicon assay was performed as described [9,10]. Briefly, DENV replicon cells were plated at 1500–2000 cells per well and 1.5–2 × 10^3^ cells per well of a 96-well dish (Corning, USA) plate the day before use. The next day, compounds were added and cells incubated at 37 °C, 5% CO_2_, in a humidified incubator for three days. The cells were the washed with PBS and Complete Promega Renilla reagent was added (Promega E2720, Madison, USA, 1 µL Renilla-Glo Substrate + 99 µL Renilla-Glo Buffer + 100 µL phenol-red-free DMEM supplemented with 10% FBS per well). Cells were lysed for 10 min at room temp and 160 µL of lysate per well was transferred to wells of a 96-well opaque white plate (Falcon 353296, Corning, USA), and luminescence was measured using a BioTek (Winooski, USA) Neo2 plate reader.

### 4.4. ZIKV Reporter Virus Assay

The ZIKV nanoluc reporter virus assay was performed as described [10,23]. Briefly, A549 Ace2 TMPRSS2 cells were plated into 96-well plates at 15,000 per well the night before use. The next day, the cells were inoculated at a multiplicity of infection (MOI) of 0.2 in medium supplemented with 1% fbs for two hours at 37 °C/5% CO_2_. The inoculum was removed, and the cells were washed once in PBS. Compounds were added in DMEM supplemented with 5% FBS. Three days later, cells were lysed and processed for luciferase as per the manufacturer’s instructions (Nano-Glo^®^ luciferase assay system, Promega N1120, Madison, WI, USA). The luciferase levels were measured using a Neo 2 plate reader (BioTek, Winooski, USA).

### 4.5. DENV CPE Assay

Huh7 cells were plated into 96-well plates at 10,000 cells per well the day before use. The cells were inoculated at an MOI of 0.1 for two hours at 37 °C/5% CO_2_. The inoculum was removed, and the cells were treated with compounds for five days. The compounds were removed, and the viability of the cells was measured using a neutral red staining assay [24]. Neutral red staining medium (40 µg/mL in cell culture medium) was prepared the night before, incubated overnight at 37 °C with shaking, and clarified with a low-speed spin (5 min, 1500 rpm, room temperature). The neutral red medium was added at 100 µL per well. The plates were incubated at 37 °C/5% CO_2_ for four hours. After incubation, the neutral red medium was removed, and the cells were washed twice in PBS. Neutral red destain buffer (50% ethanol, 49% H_2_O, and 1% glacial acetic acid) was added at 100 µL per well and the plates were read at 540 nm in a Molecular Device (San Jose, CA, USA) M5e plate reader.

## 5. Conclusions

The drug combination strategy described in this article presents several advantages. Combining two antivirals compared to a single antiviral drug may result in multiple benefits, such as an increase in efficacy while using lower doses, higher compliance, a lower toxicity, a higher resilience to drug resistance development, and a synergistic and broad-spectrum antiviral effect. Our previous studies demonstrated the potentiation of FAV, a purine nucleobase analogue when combined with 6MMPR and a de novo purine nucleotide biosynthesis inhibitor, leading to a broad-spectrum and synergistic antiviral effect. In this article, we expanded this study to include pyrimidines, but we did not observe a potentiation effect. However, we found a novel antiviral combination with ribavirin nucleobase potentiated by 6MMPR prodrug, a potentially significant result and alternative to the broad-spectrum antivirals ribavirin (nucleoside) or FAV. Further studies are needed with additional pyrimidine and purine nucleobases and related de novo biosynthesis inhibitors to confirm these results. Overall, this research advances knowledge on drug combination strategies and anti-dengue and broad-spectrum antivirals which are highly relevant to the scientific community for pandemic preparedness and public health in general.

## Figures and Tables

**Table 1:** Potency (EC_50_) and toxicity (CC_50_) of pyrimidine nucleobase/nucleoside analogues and pyrimidine de novo nucleotide biosynthesis inhibitors.

Compound	EC_50_ (μM)	CC_50_ (μM)
2-thiouracil (2SU)	>50	>50
2-thiouridine	>50	>50
5-fluorouracil (5FU)	0.74	0.93
*N*-hydroxy-cytidine (NOHC)	>100	>100
Molnupiravir	6.2	53
*N*-amino-cytidine (NAC)	>100	>100
6-azauridine	1.9	8.8
Brequinar	3.0	4.5

**Table 2:** Potency (EC_50_) and toxicity (CC_50_) of purine nucleobase analogues and purine de novo nucleotide biosynthesis inhibitors.

Compound	EC_50_ (μM)	CC_50_ (μM)
Favipiravir (FAV)	114	>500
T-1105	13	>500
Ribavirin nucleobase	5.7	>100
6MMPR	0.21	0.21
Prdg 1	0.26	2.6
Prdg 2	2.5	5.4
Prdg 3	2.3	2.4

**Table 3:** Purine nucleobase combinations with purine de novo purine nucleotide biosynthesis inhibitors.

Compounds	EC_50_ (μM)	CC_50_ (μM)
Favipiravir (Fav)	128	>500
+6MMPR 0.065 μM	63	>500
+Prdg 1 0.09 μM	51	>500
+Prdg 2 1.5 μM	47	>500
Favipiravir (Fav)	90	>500
+Prdg 3 1.4 μM	46	>500
T-1105	15	>167
+6MMPR 0.065 μM	11	>167
+Prdg 1 0.09 μM	8.3	>167
+Prdg 2 1.5 μM	10	>167
+Prdg 3 1.4 μM	9.6	>167
Ribavirin nucleobase	5.7	>50
+6MMPR 0.065 μM	3.4	>50
+Prdg 1 0.09 μM	2.3	>50
+Prdg 2 1.5 μM	0.88	>50
+Prdg 3 1.4 μM	2.6	>50

## Data Availability

Additional data are available in the Supplementary Materials.

## Supplementary Materials

The following supporting information can be downloaded at: https://www.mdpi.com/article/10.3390/molecules30020210/s1.
