# Supplementary material for: Purine but Not Pyrimidine De Novo Nucleotide Biosynthesis Inhibitors Strongly Enhance the Antiviral Effect of Corresponding Nucleobases Against Dengue Virus
Source: Molecules. 2025 Jan 7;30(2):210. doi: 10.3390/molecules30020210 (PMC11767801; doi:10.3390/molecules30020210)
Supplement: Supplementary file 1 [file molecules-30-00210-s001.zip › molecules-3346398-supplementary.pdf]

Supporting Information for

Purine but Not Pyrimidine *De-Novo*-Nucleotide-Biosynthesis Inhibitors Strongly Enhance the Antiviral Effect of Corresponding Nucleobases Against Dengue Virus

Laurent F. Bonnac*, Christine D. Dreis, Madhu Rai, Robert J. Geraghty*

[a]*Center for Drug Design, College of Pharmacy, University of Minnesota, Minneapolis, USA*

Table of Contents:

SI-1 Materials and Methods, Experimental Procedures
SI-2 ^{1}H,^{13}C NMRs
SI-3 Biological evaluation

SI-1 Materials and Methods, Experimental Procedures

All commercial reagents were used as provided unless otherwise indicated. An anhydrous solvent-dispensing system (J.C. Meyer) using two packed columns of neutral alumina was used for drying THF, Et_2O, and CH_2Cl_2, whereas two packed columns of molecular sieves were used to dry DMF. Solvents were dispensed under argon. Flash chromatography was performed with Ultra Pure silica gel (SiliCycle) or with RediSep Rf silica gel columns on a Teledyne ISCO CombiFlash Rf system using the solvents as indicated. All reactions were performed under a dry atmosphere of argon unless otherwise specified. Indicated reaction temperatures refer to the reaction bath, while room temperature (rt) is noted as 25°C. Commercial grade reagents and anhydrous solvents were used as received from vendors, and no attempts were made to purify or dry these components further. Removal of solvents under reduced pressure was accomplished with a Buchi rotary evaporator at approximately 28 mmHg pressure using a Teflon-linked KNF vacuum pump. Thin layer chromatography was performed using either 1 in. × 3 in. Anal Tech No. 02521 or Merck 60 F254 silica gel plates with fluorescent indicator using appropriate solvent mixtures. Visualization of TLC plates was made by observation with either short wave UV light (254 nm lamp) or 10% sulfuric acid in ethanol. Nuclear magnetic resonance spectra were recorded on a Varian 600 MHz or Bruker 400 spectrometer with Me4Si or signals from residual solvent as the internal standard for 1H. Chemical shifts are reported in ppm, and signals are described as s (singlet), d (doublet), t (triplet), q (quartet), m (multiplet), br s (broad singlet), and dd (double doublet). Values given for coupling constants are of first order. Mass spectroscopic analyses were performed either using positive mode electron spray ionization (ESI) on a Varian ProStar LC-MS with a 1200L quadrupole mass spectrometer or using positive mode atmospheric pressure chemical ionization (APCI) on a Shimadzu LC-MS system. High performance liquid chromatography (HPLC) purity analysis was conducted using a Varian Pro Star HPLC system with a binary solvent system A and B using a gradient elution [A, H2O with 0.1% trifluoroacetic acid (TFA); B, CH_3CN with 0.1% TFA] and flow rate = 1 mL/min, with UV detection at 254 nm. All final compounds were purified to ≥95% purity, and these purity levels were measured by a Varian Pro Star HPLC system. Three different Varian Pro Star HPLC methods were used to establish compound purity. HPLC Method A: Phenomenex Luna C18(2) column (4.6 mm × 250 mm); mobile phase, A = H2O with 0.1% TFA and B = CH_3CN with 0.1% TFA; gradient 10−95 % B (0.0−10 min; hold for 6 min); UV detection at 254 nm. HPLC Method B: SunFire C18 column (4.6 mm × 250 mm); mobile phase, A = H2O with 0.1% TFA and B = CH_3CN with 0.1% TFA; gradient 10−100 % B (0.0−20 min; hold for 5 min); UV detection at 254 nm. HPLC Method C: SunFire C18 column (4.6 mm × 250 mm); mobile phase, A = H_2O with 0.1% TFA and B = CH_3CN with 0.1% TFA; gradient 0−100 % B (0.0−15 min; hold for 5 min)); UV detection at 254 nm.

Prdg 1

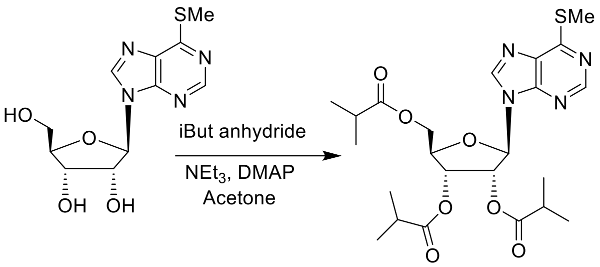

6-methylmercaptopurine riboside (6MMPR) (1eq, 0.330g, 1.1mmol) is suspended in acetone (6mL). A catalytic amount of dimethylaminopyridine (DMAP) and triethylamine (NEt_3) (3.3eq, 0.369g, 3.65mmol) are added and the reaction mixture is cooled to 0 ^{o}C with an ice bath. Isobutyric anhydride (3.3eq, 0.577g, 3.65mmol) is added, and the reaction mixture is stirred at room temperature overnight. The mixture is then dissolved in 200mL of dichloromethane and wash with water (3x100mL). The organic phase is concentrated and purified on silica gel chromatography (DCM, MeOH 0 to 2 %), Rf= 0.4 (DCM, MeOH 2%), colorless thick oil, yield 86%. ^{1}H NMR (400 MHz, $CDCl_3$) δ 8.71 (s, 1H), 8.11 (s, 1H), 6.20 (d, *J* = 5.5 Hz, 1H), 5.89 (t, *J* = 5.5 Hz, 1H), 5.64 (dd, *J* = 5.6, 4.3 Hz, 1H), 4.55 – 4.29 (m, 3H), 2.71 (s, 3H), 2.67 – 2.42 (m, 3H), 1.36 – 0.87 (m, 18H).^{13}C NMR (101 MHz, $CDCl_3$) δ 176.91, 176.02, 175.77, 162.68, 152.57, 148.31, 141.25, 132.26, 86.86, 81.25, 73.63, 70.93, 63.47, 34.32, 34.20, 34.06, 19.41, 19.30, 19.27, 19.23, 19.16, 19.06, 12.26. HRMS (ESI^{+}): m/z calculated for $C_{23}H_{33}N_4O_7S+$ $[M+H]^+$ 5092064, found 509.2060

Prdg 2

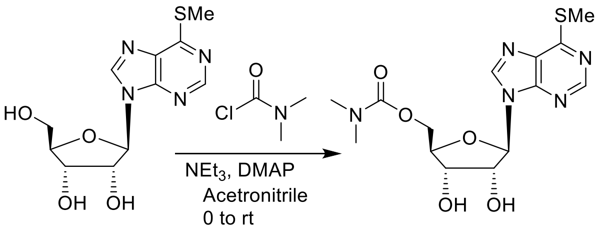

6-methylmercaptopurine riboside (6MMPR) (1eq, 0.330g, 1.1mmol) is suspended in acetonitrile (18mL). A catalytic amount of dimethylaminopyridine (DMAP) and triethylamine (NEt_3) (1.1eq, 0.123g, 1.21mmol) are added and the reaction mixture is cooled to 0 ^{o}C with an ice bath. The acyl chloride (1.1eq, 0.130g, 1.21mmol) is added dropwise over 10 minutes. The mixture is then stirred at room temperature for 72 hours. The mixture is then dissolved in 300mL of dichloromethane and wash with water (3x100mL). The organic phase is concentrated and purified on silica gel chromatography (DCM, MeOH 0 to 5 %), Rf= 0.2 (DCM, MeOH 5%), white paste, yield 25%. ^{1}H NMR (400 MHz, MeOD) δ 8.69 (s, 1H), 8.60 (s, 1H), 6.11 (d, *J* = 6.7 Hz, 1H), 5.29 (dd, *J* = 5.3, 2.5 Hz, 1H), 4.97 (dd, *J* = 6.8, 5.3 Hz, 1H), 4.30 (q, *J* = 2.7 Hz, 1H), 4.02 – 3.65 (m, 2H), 3.06 (s, 3H), 2.95 (s, 3H), 2.72 (s, 3H). ^{13}C NMR (101 MHz, MeOD) δ

163.33, 157.29, 152.60, 148.63, 144.06, 132.55, 90.36, 85.94, 75.75, 74.26, 62.84, 36.53, 36.17, 11.58. HRMS (ESI$^+$): m/z calculated for $C_{14}H_{20}N_5O_5S+$ $[M+H]^+$ 370.1180, found 370.1181

Prdg 3

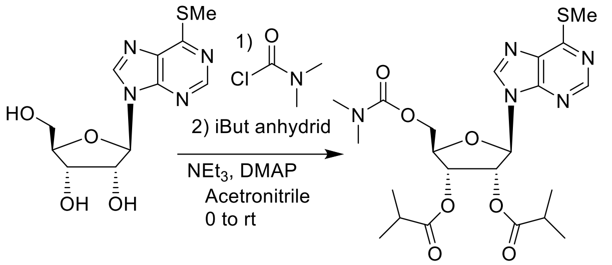

6-methylmercaptopurine riboside (6MMPR) (1eq, 0.330g, 1.1mmol) is suspended in acetonitrile (18mL). A catalytic amount of dimethylaminopyridine (DMAP) and triethylamine (NEt_3) (1.1eq, 0.123g, 1.21mmol) are added and the reaction mixture is cooled to 0 ^oC with an ice bath. The acyl chloride (1.1eq, 0.130g, 1.21mmol) is added dropwise over 10 minutes. The mixture is then stirred at room temperature for 72 hours. The mixture is cooled to 0 ^oC with an ice bath and isobutyric anhydride is added. The mixture is then stirred at room temperature for 48 hours. The mixture is then dissolved in 300mL of dichloromethane and wash with water (3x100mL). The organic phase is concentrated and purified on silica gel chromatography (Ethyl acetate, MeOH 0 to 5 %), Rf= 0.7 (EA, MeOH 5%), colorless thick oil, yield 35%. ^1H NMR (400 MHz, CDCl3) δ 8.73 (s, 1H), 8.15 (s, 1H), 6.26 (d, *J* = 5.6 Hz, 1H), 5.81 (t, *J* = 5.6 Hz, 1H), 5.64 (dd, *J* = 5.6, 4.3 Hz, 1H), 4.49 – 4.32 (m, 3H), 2.89 (s, 3H), 2.85 (s, 3H), 2.73 (s, 3H), 2.62 (dt, *J* = 11.5, 7.0 Hz, 2H), 1.29 – 1.13 (m, 12H). ^{13}C NMR (101 MHz, $CDCl_3$) δ 176.35, 175.33, 161.96, 154.13, 151.77, 147.90, 140.80, 131.61, 86.14, 80.72, 74.18, 70.65, 63.00, 36.48, 35.76, 33.75, 33.69, 18.83, 18.74, 18.69, 18.52, 11.85. HRMS (ESI$^+$): m/z calculated for $C_{22}H_{32}N_5O_7S+$ $[M+H]^+$ 510.2017, found 510.2010

SI-3 ^{1}H,^{13}C NMRs

Prdg 1

^{1}H NMR (400 MHz, $CDCl_3$) δ 8.71 (s, 1H), 8.11 (s, 1H), 6.20 (d, *J* = 5.5 Hz, 1H), 5.89 (t, *J* = 5.5 Hz, 1H), 5.64 (dd, *J* = 5.6, 4.3 Hz, 1H), 4.55 – 4.29 (m, 3H), 2.71 (s, 3H), 2.67 – 2.42 (m, 3H), 1.36 – 0.87 (m, 18H).^{13}C NMR (101 MHz, $CDCl_3$) δ 176.91, 176.02, 175.77, 162.68, 152.57, 148.31, 141.25, 132.26, 86.86, 81.25, 73.63, 70.93, 63.47, 34.32, 34.20, 34.06, 19.41, 19.30, 19.27, 19.23, 19.16, 19.06, 12.26.

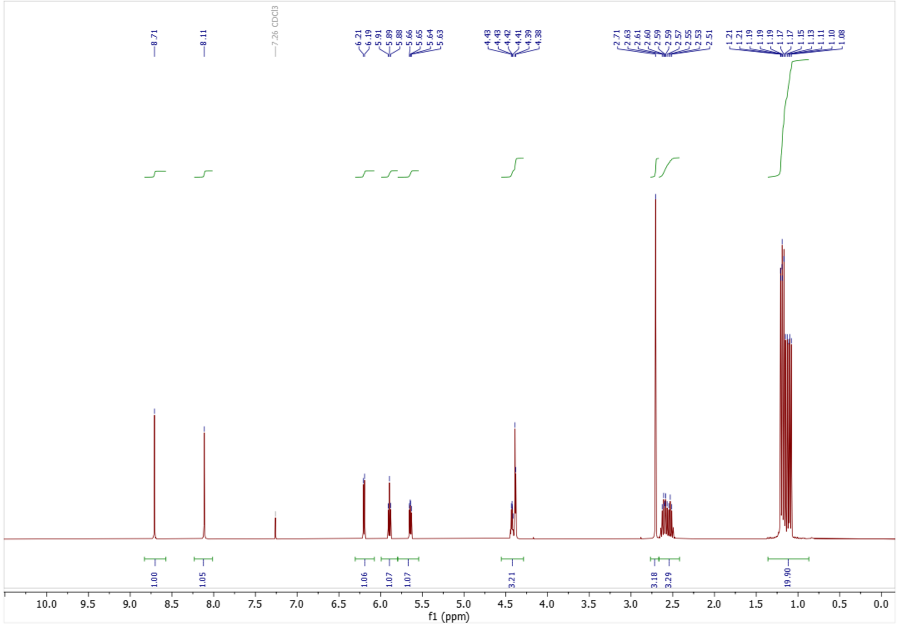

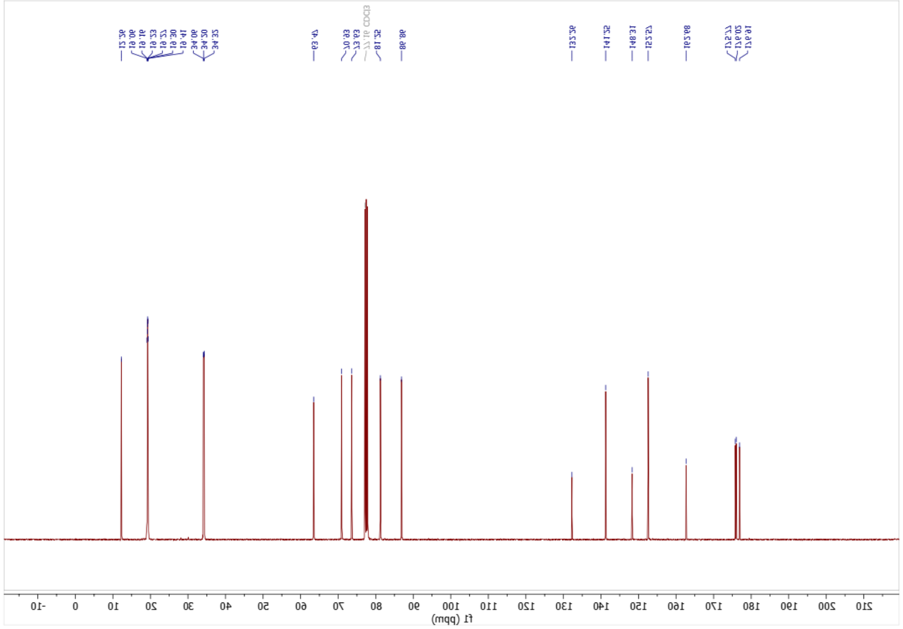

Prdg 2

^{1}H NMR (400 MHz, MeOD) δ 8.69 (s, 1H), 8.60 (s, 1H), 6.11 (d, *J* = 6.7 Hz, 1H), 5.29 (dd, *J* = 5.3, 2.5 Hz, 1H), 4.97 (dd, *J* = 6.8, 5.3 Hz, 1H), 4.30 (q, *J* = 2.7 Hz, 1H), 4.02 – 3.65 (m, 2H), 3.06 (s, 3H), 2.95 (s, 3H), 2.72 (s, 3H). ^{13}C NMR (101 MHz, MeOD) δ 163.33, 157.29, 152.60, 148.63, 144.06, 132.55, 90.36, 85.94, 75.75, 74.26, 62.84, 36.53, 36.17, 11.58.

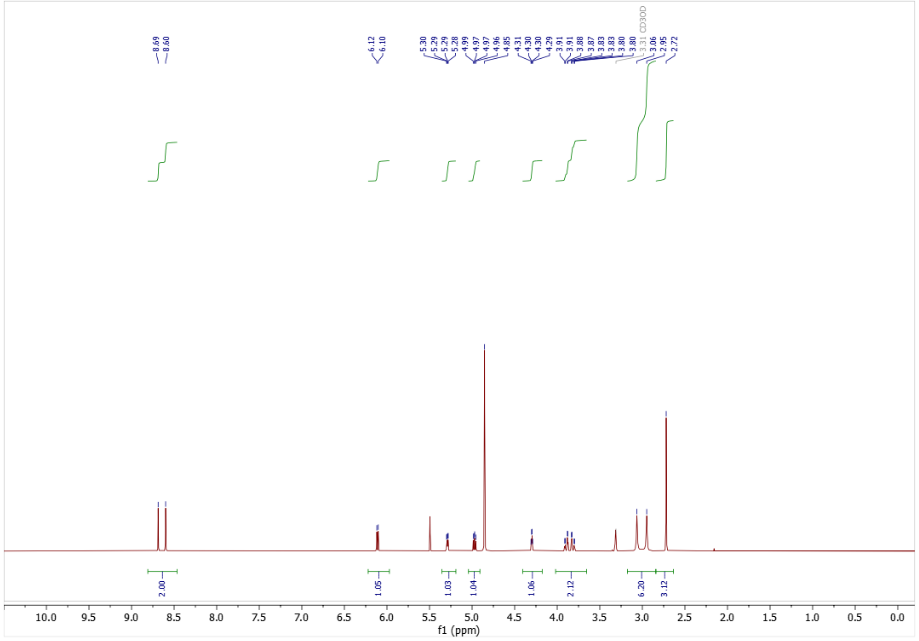

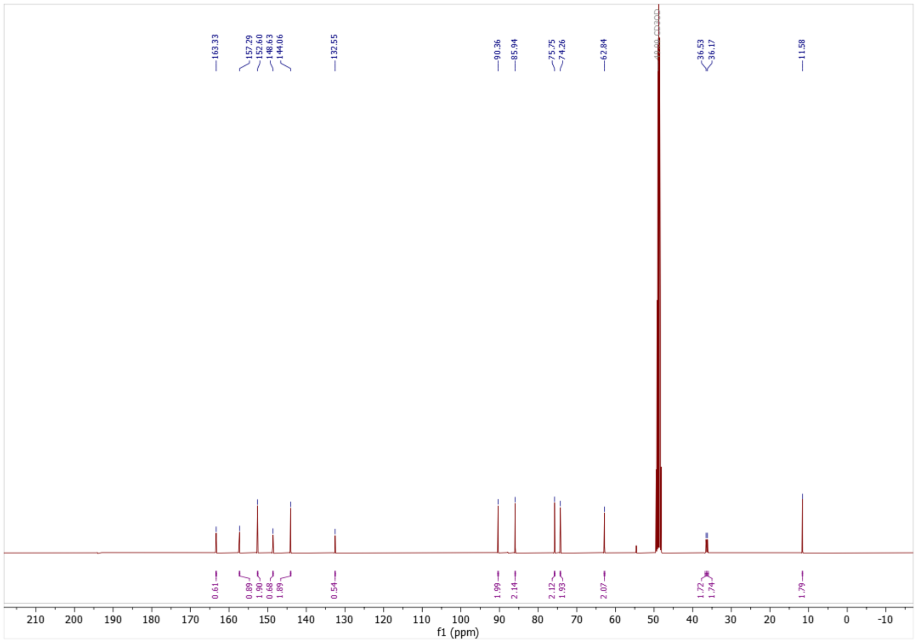

Prdg 3

^{1}H NMR (400 MHz, CDCl3) δ 8.73 (s, 1H), 8.15 (s, 1H), 6.26 (d, *J* = 5.6 Hz, 1H), 5.81 (t, *J* = 5.6 Hz, 1H), 5.64 (dd, *J* = 5.6, 4.3 Hz, 1H), 4.49 – 4.32 (m, 3H), 2.89 (s, 3H), 2.85 (s, 3H), 2.73 (s, 3H), 2.62 (dt, *J* = 11.5, 7.0 Hz, 2H), 1.29 – 1.13 (m, 12H). ^{13}C NMR (101 MHz, $CDCl_3$) δ 176.35, 175.33, 161.96, 154.13, 151.77, 147.90, 140.80, 131.61, 86.14, 80.72, 74.18, 70.65, 63.00, 36.48, 35.76, 33.75, 33.69, 18.83, 18.74, 18.69, 18.52, 11.85.

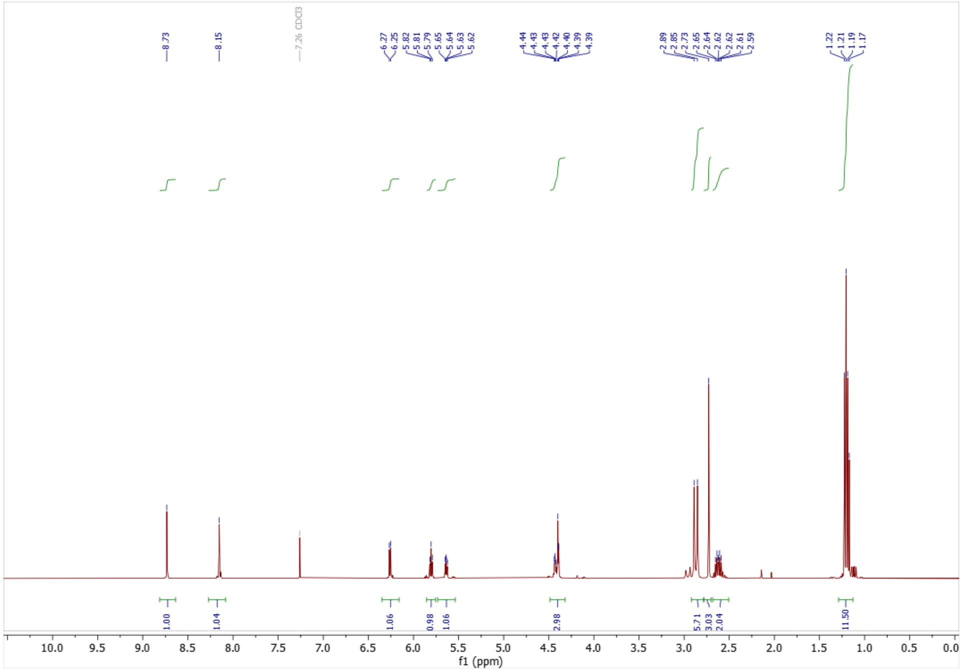

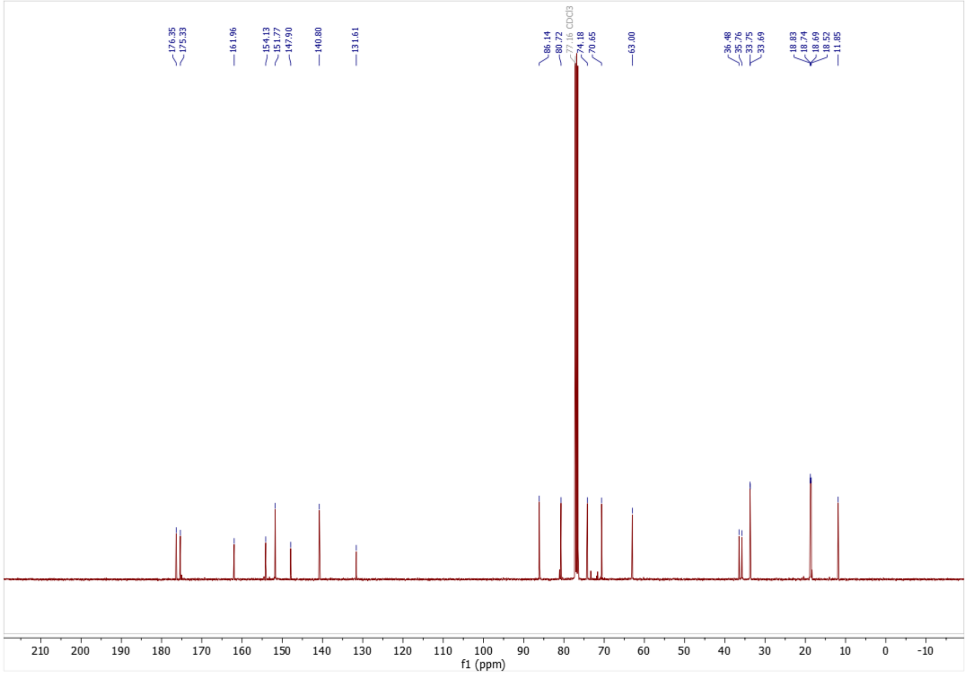

SI-3 Biological evaluations

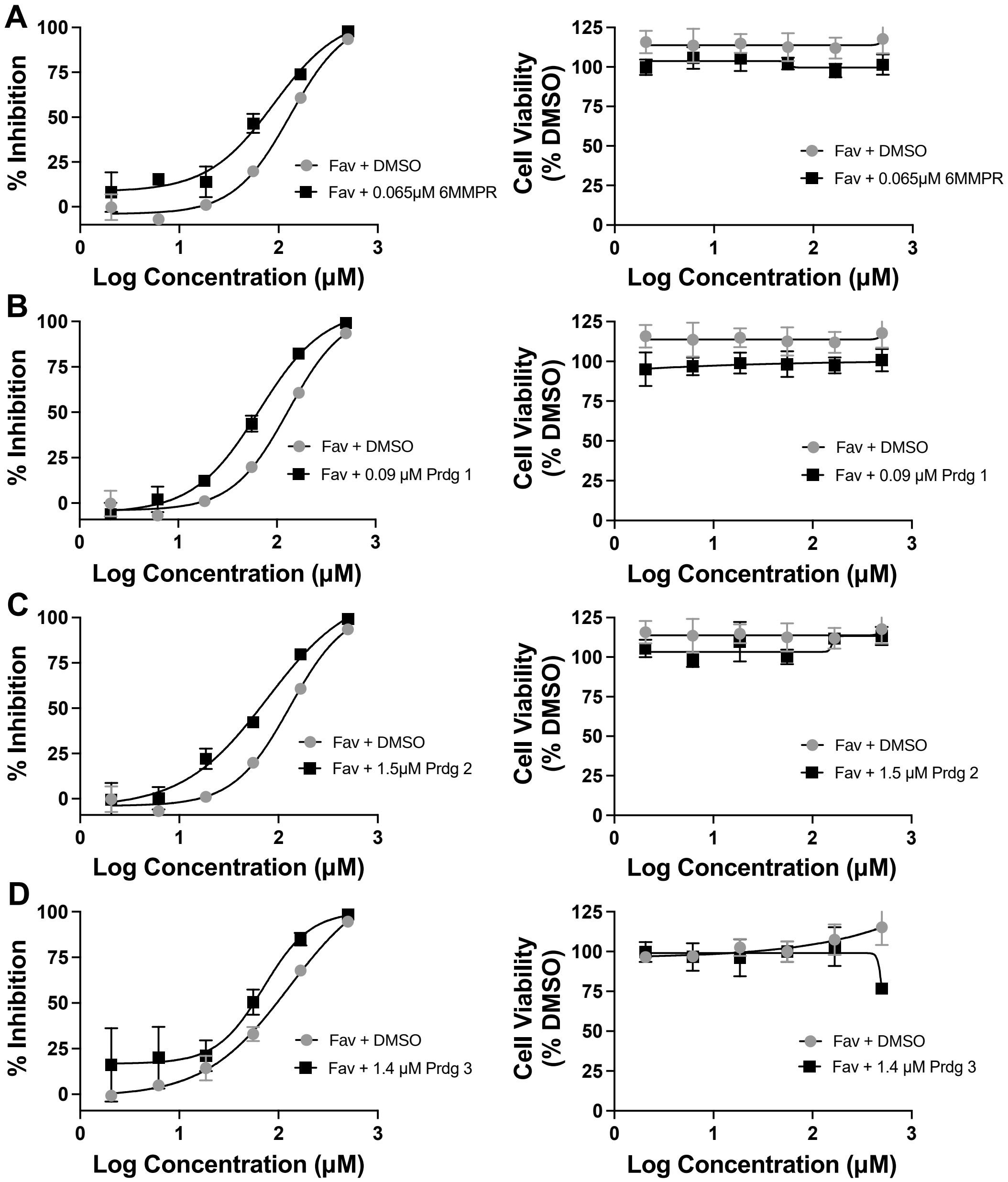

Figure S1. Favipiravir (Fav) and 6MMPR prodrug combinations and effects on DENV replicon replication. DENV BHK replicon cells were treated with compounds indicated and three days later evaluated for luciferase activity (left graph) and cell viability (right graph). The results for Fav plus vehicle (DMSO) were plotted with: **A.** Fav with 0.065 μM 6MMPR. **B.** Fav with 0.09 μM Prdg 1. **C.** Fav with 1.5 μM Prdg 2. **D.** Fav with 1.4 μM Prdg 3. Fav doses ranged from 500 to 2 μM. Results were plotted in GraphPad Prism and EC_{50}/CC_{50} values listed in **Table 3**.

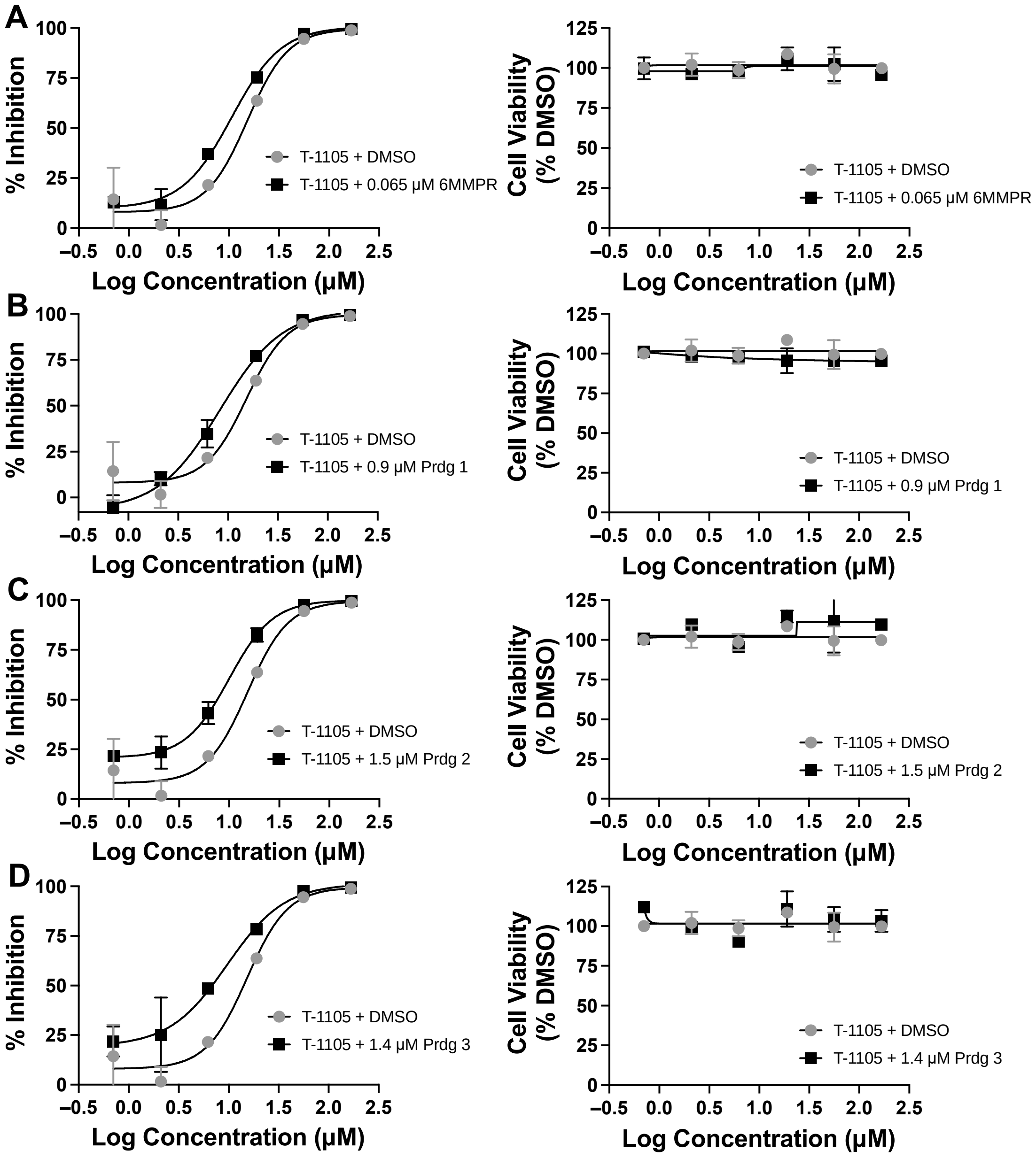

Figure S2. T-1105 and 6MMPR prodrug combinations and effects on DENV replicon replication. DENV BHK replicon cells were treated with compounds indicated and three days later evaluated for luciferase activity (left graph) and cell viability (right graph). The results for T-1105 plus vehicle (DMSO) were plotted with: **A.** T-1105 with 0.065 µM 6MMPR. **B.** T-1105 with 0.09 µM Prdg 1. **C.** T-1105 with 1.5 µM Prdg 2. **D.** T-1105 with 1.4 µM Prdg 3. T-1105 doses ranged from 167 to 0.7 µM. Results were plotted in GraphPad Prism and EC_{50}/CC_{50} values listed in **Table 3**.

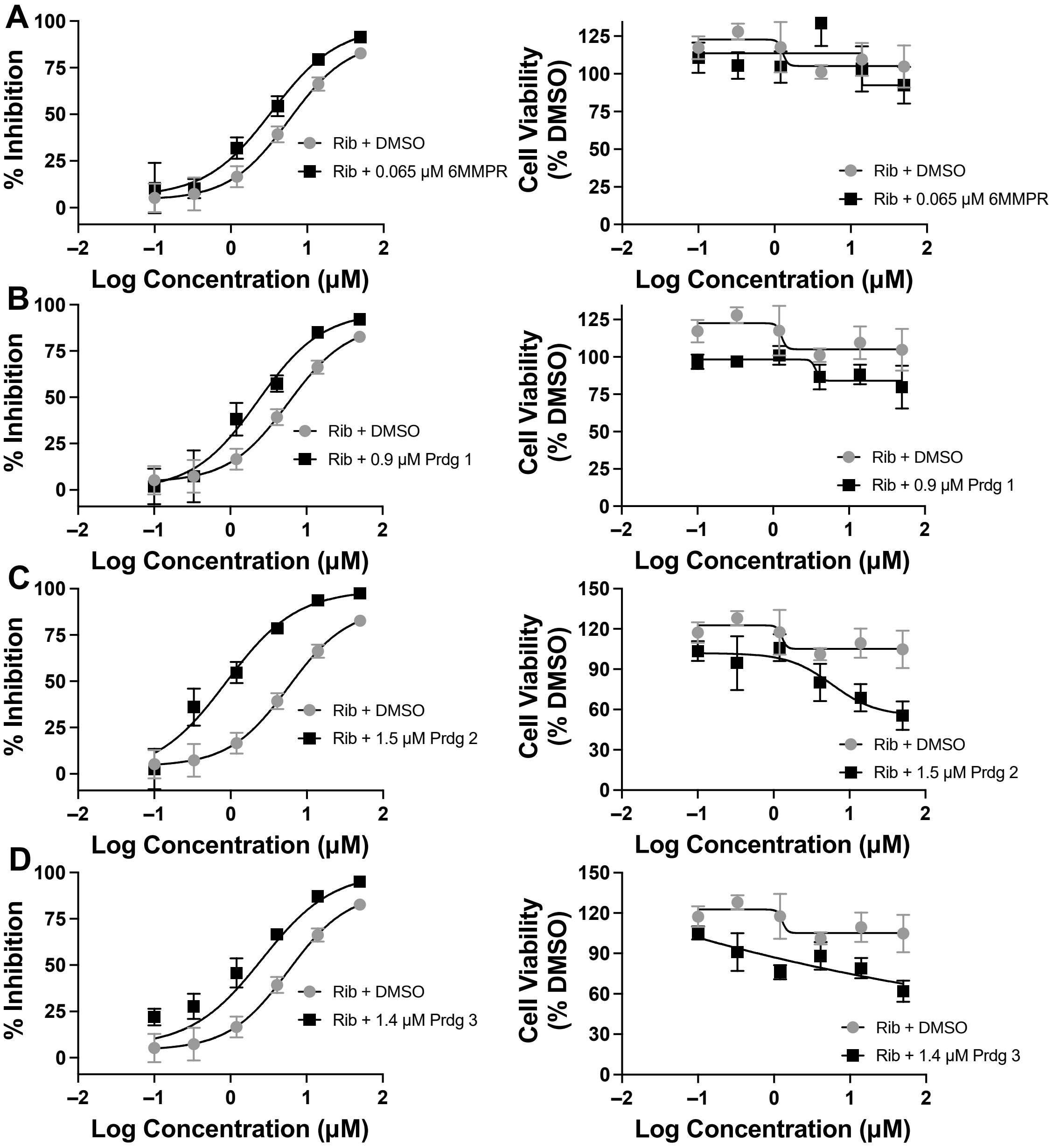

Figure S3. Ribavirin base (Rib) and 6MMPR prodrug combinations and effects on DENV replicon replication. DENV BHK replicon cells were treated with compounds indicated and three days later evaluated for luciferase activity (left graph) and cell viability (right graph). The results for Rib plus vehicle (DMSO) were plotted with: **A.** Rib with 0.065 μM 6MMPR. **B.** Rib with 0.09 μM Prdg 1. **C.** Rib with 1.5 μM Prdg 2. **D.** Rib with 1.4 μM Prdg 3. Rib doses ranged from 50 to 0.1 μM. Results were plotted in GraphPad Prism and EC_{50}/CC_{50} values listed in **Table 3.**
